# Comprehensive characterization of four different populations of human mesenchymal stem cells as regards their immune properties, proliferation and differentiation

**DOI:** 10.3892/ijmm.2014.1821

**Published:** 2014-06-25

**Authors:** XIUYING LI, JINPING BAI, XIAOFENG JI, RONGGUI LI, YALI XUAN, YIMIN WANG

**Affiliations:** 1 The Central Laboratory, China-Japan Union Hospital, Jilin University, Changchun, Jilin 130033, P.R. China; 2 Department of Orthopedics, China-Japan Union Hospital, Jilin University, Changchun, Jilin 130033, P.R. China; 3 Department of Pathology, Jilin University, The Key Laboratory of Pathobiology, Ministry of Education, Changchun, Jilin 130021, P.R. China; 4 Jilin Zhongke Bio-engineering, Co., Ltd., Changchun, Jilin 130012, P.R. China

**Keywords:** bone marrow, immunomodulation, lymphocytes, mesenchymal stem cells, Wharton’s Jelly

## Abstract

In the present study, we compared mesenchymal stem cells (MSCs) derived from 4 different sources, human bone marrow (BM), adipose tissue (AT), umbilical cord Wharton’s Jelly (WJ) and the placenta (PL), in order to determine which population of MSCs displayed the most prominent immunosuppressive effects on phytohemagglutinin-induced T cell proliferation, and which one had the highest proliferative and differentiation potential. MSC and T lymphocyte co-culture (mixed culture) was used to determine whether the MSCs inhibit T cell proliferation, as well as which population of MSCs has the strongest inhibitory ability. The expression of immune-related genes was analyzed by RT-PCR and RT-qPCR. The proliferation and differentiation potential of the MSCs were determined using standard methods. Following MSC and T cell co-culture, mitogen-induced T cell proliferation was effectively suppressed by all 4 populations of MSCs. This occurred through soluble factors rather than direct contact inhibition. Among the 4 populations of MSCs, the WJ-MSC has the strongest suppression effects. On immune related genes, WJ-MSC has the weakest expression of MHC II genes, TLR4, TLR3, JAG1, NOTCH2 and NOTCH3. To compare the proliferation potential, WJ-MSCs showed the most rapid growth rate followed by the AT-, PL- and BM-MSCs. As regards differentiation potential, the WJ-MSCs had the strongest osteogenetic ability followed by PL, AT and BM-MSC. AT-MSC has the strongest adipogenetic ability followed by the WJ-, BM- and PL-MSCs. These data indicated that the WJ-MSCs had the strongest immunomodulatory and immunosuppressive potential. In light of these observations, we suggest that WJ-MSCs are the most attractive cell population for use in immune cellular therapy when immunosuppressive action is required.

## Introduction

Mesenchymal stem cells (MSCs) are multipotent stromal cells that have been isolated from both adult and fetal tissues and are defined as adherent, fibroblast-like cells. In the early 1970s, Friedenstein *et al* described the existence of multipotent mesenchymal cells in mouse bone marrow (BM) with the ability to form colonies [fibroblast colony-forming units (CFU-F)] and differentiate into adipocytes, chondrocytes and osteocytes (1). It was only 20 years later that Caplan defined the terminology, MSCs (2). Subsequently, approximately 10 years later, MSCs were finally identified in human adult BM (3,4). MSCs are initially isolated and characterized from BM, but can be also obtained from other sources, such as the amniotic membrane, skin, hair follicles, dental pulp, adipose tissue (AT), cord blood, umbilical cord Wharton’s jelly (WT), the endometrium, amniotic fluid, fetal liver, the placenta (PL) and the synovium (5). Among these sources, AT, WJ from the umbilical cord and PL are considered to be valuable alternatives to BM as a rich source of MSCs (6,7).

MSCs possess 2 major properties, a self-renewal ability and the potential for multilineage differentiation. MSCs can differentiate into a variety of cell types, including osteoblasts, chondrocytes, adipocytes and myocytes (8–12). It has been reported that the most important characteristics of MSCs are their potential for differentiation into bone and cartilage cell lineages (13,14). The differential ability of MSCs raises the hope for treating some types of bone or cartilage injuries which can be treated by general medication practices (15,16). *In vitro* and *in vivo* studies have also demonstrated that MSCs can differentiate into cells of non-mesodermal origin, such as neurons, skin and gut epithelial cells, hepatocytes and pneumocytes (17).

It has been demonstrated that MSCs have both immunosuppressive and immunomodulatory functions (18–20). Although, the mechanisms underlying the behavior of MSCs during an immune response and their immunomodulatory effects remain unclear, tissue-derived MSCs have potent immunomodulatory properties and suppress T lymphocyte, B lymphocyte and natural killer (NK) cell functions (21–24). Members of the human leukocyte antigen (HLA) family and immunoregulatory factors are of importance in determining the nature of the response generated by MSCs and T lymphocyte interactions. Thus, establishing and comparing the immunological profiles of MSCs isolated from different types of tissue may facilitate the determination of the best immune-privileged MSCs for clinical therapy.

## Materials and methods

### Isolation and expansion of MSCs

MSCs were isolated from 4 different sources: BM, AT, WJ and PL tissue. The BM and AT samples were obtained from healthy volunteer donors, while the WJ and PL samples were from tissue following normal caesarean birth. All individuals provided written informed consent and the study was approved by the Ethics Committee of the China-Japan Union Hospital, Jilin University, Changchun, China. The age range of the donors was as follows: the BM was from individuals aged 18 to 43 years, the AT was from individuals aged 23 to 50 years, and the WJ and PL tissue were from individuals aged 23 to 38 years. The MSCs derived from BM, AT, WJ and PL were isolated according to previously described methods (25–27) with some modifications. The enzymatic digestion method was used to isolate the MSCs from the tissues. Briefly, collagenase and hyaluronidase were used to digest the umbilical cord after the outside skin was removed. The PL and AT were digested by collagenase only. BM-MSCs were obtained by BM adherence culture. The MSCs were cultured in α-MEM supplemented with 10% FBS (Invitrogen Australia Pty Ltd., Mount Waverley, Victoria, Australia). The culture was maintained at 37°C with saturated humidity and 5% CO_2_. After 48 h, the non-adherent cells were removed by washing, and the medium was changed twice a week. The MSCs were subcultured at 80% confluence following treatment with 0.05% trypsin and 0.02% EDTA (Sigma, St. Louis, MO, USA) for 3 min at 37°C. The cells were washed and harvested by centrifugation at 1000 rpm for 5 min, then replanting at a lower density (1,000 cells/cm^2^).

### Flow cytometric characterization of MSCs

The MSCs were removed from the culture flasks by incubation in 0.05% trypsin-EDTA at 37°C and then washed twice with PBS. The cell suspensions were then incubated with antibodies against CD44-phycoerythrin (PE), CD73-PE, CD14-PE, CD34-PE, CD90-fluorescein isothiocyanate (FITC), CD45-FITC and CD105-PerCP (BD Biosciences, Franklin Lakes, NJ, USA) and protected from light for 30 min at 4°C. Following incubation, the cells were washed twice with PBS. The fluorescence intensity of the cells was evaluated using a flow cytometer (FACScan; BD Biosciences) and the data were analyzed using CellQuest software (BD Biosciences).

### MSC differentiation potential

#### Adipogenesis

A total of 5x10^4^ MSCs/cm^2^ was seeded into a 12-well plate and incubated in MSC growth medium (α-MEM with 10% FBS) at 37°C in a humidified atmosphere with 5% CO_2_ for a minimum of 2 h or up to 4 days (to near or complete confluence) before re-seeding with adipogenic differentiation medium (StemPro^®^ Adipogenesis Differentiation kit; Invitrogen Australia Pty Ltd.). The MSCs will continue to undergo limited expansion as they differentiate under adipogenic conditions. The cultures were re-fed every 3–4 days. After 21 days under differentiating conditions, the medium was removed from the wells, and the cells were rinsed once with PBS, and fixed with 4% formaldehyde solution for 30 min. After fixation, the cells were rinsed twice with PBS, stained with Oil Red O (Sigma) for 15 min, rinsed twice with PBS and visualized under a light microscope (Olympus, Tokyo, Japan). Images were captured for qualitative and quantitative analyses. Fifteen fields at x400 magnification under the microscope were randomly selected and the stained cell and total cell numbers were counted. The sample number was 3 each for all 4 populations of MSCs.

#### Osteogenesis

The MSCs were seeded into a 12-well plate at 5x10^4^ cells/cm^2^. The cells were fed in α-MEM with 10% FBS and incubated at 37°C in a humidified atmosphere with 5% CO_2_. Osteogenic differentiation medium (StemPro^®^ Osteogenesis Differentiation kit; Invitrogen Australia Pty Ltd.) was added to the cells the second day after the cells attached to the bottom of the petri dish. MSCs will undergo limited expansion during differentiation under osteogenic conditions. The cultures were re-fed every 3–4 days. On day 35, the medium was removed from the 12-well plate and the cells were rinsed twice with PBS. The cells were fixed with 4% formaldehyde solution for 30 min. After fixation, the cells were rinsed with distilled water and stained with 2% Alizarin Red S solution (Alizarin Staining kit; Genmed, Shanghai, China) for 5 min. The cells were rinsed 3 times with distilled water and visualized under a light microscope (Olympus). Images were captured for qualitative or quantitative analyses. Bone nodule numbers of a total of 3 wells were counted for each sample. Three samples from each population of MSCs were analyzed.

### Proliferative potential of MSCs

The 4 populations of MSCs beginning from passage 1 were trypsinized from the culture discs before plating into the 6-well plates at a density of 10^5^/well. Each population of MSCs was seeded in triplicate. Every 4 days, the cells were harvested with trypsin/EDTA and counted using a hemacytometer for up to 8 passages. The mean value of the cell number counts was calculated from 7 tissue samples from each cell population and the mean population doubling time was obtained for each passage according to the following formula: population doubling time = T x lg2/(lgNt - lgN0), where T is the culture time, N0 is the initial cell number and Nt is the harvested cell number.

### Reverse transcription PCR (RT-PCR)

Total cellular RNA was extracted using TRIzol reagent (Invitrogen Life Technologies, Grand Island, NY, USA) according to the manufacturer’s instructions. RNA integrity was electrophoretically verified by ethidium bromide staining and a OD260/OD280 nm absorption ratio (OD >1.9). Total RNA (500 ng) was reverse transcribed using AWV reverse trancriptase, oligo(dT)15 (~20 mer, Takara, Dalian, China) according to the manufacturer’s instructions. No amplification control (NAC) was included without adding reverse transcriptase. Reactions were incubated at 42°C for 30 min, then 95°C for 5 min and 5°C for 5 min.

Twenty five nanograms of cDNA was used in a 25-μl reaction volume with 0.15 μl Takara Ex Taq HS (Takara), 5 μM of gene-specific forward and reverse primers (Sangon Biotech, Shanghai, China), 5 μl 5X PCR buffer and sterile water. Reactions were incubated at 94°C for 30 sec, 56°C for 30 sec and 72°C for 30 sec for total 30 cycles.

### Quantitative reverse transcription PCR (RT-qPCR)

RT-qPCR was carried out to assess the expression of genes, including HLA-DMA, HLA-DRA, HLA-DPB1, JAG1, TLR4, TLR3, NOTCH2 and NOTCH3 in the MSCs derived from the 4 different tissue sources. Primers were designed using primer3 input (http://flypush.imgen.bcm.tmc.edu/primer/primer3_www.cgi) and synthesized with the temperature at 60°C (Sangon Biotech; Table I). RNA extraction and reverse transcription were carried out as described above. RT-qPCR was carried out using the ABI PRISM 7900 Sequence Detection System (Perkin-Elmer/Applied Biosystems, Foster City, CA, USA). Twenty five nanograms of cDNA was used in the qRT-PCR reactions with SYBR-Green PCR Master Mix (Applied Biosystems, Warrington, UK) and 5 μM of gene-specific forward and reverse primers (Sangon Biotech). All PCR products demonstrated a single band by a dissociation curve and gel electrophoresis. The thermal cycler parameters for the amplification of these genes were as follows: 1 cycle at 95°C for 10 min followed by 40 cycles at 95°C for 15 sec, 55°C for 15 sec and 72°C for 30 sec. The 18S RNA gene was used to normalize the cDNA amounts used in RT-qPCR. Amplification using 18S RNA primers consistently yielded similar CT levels among all types of tissue. No template control (NTC) and NAC were included in each reaction. Gene expression was presented using a modification of the 2^−ΔΔCt^ method, first described by Livak and Schmittgen in the PE Biosystems Sequence Detector User Bulletin 2 (28).

### Co-culture of MSCs and T lymphocytes

The co-culture of the MSCs and T cells was carried out using 24-well plates (contact culture) and the Transwell system (isolated culture) in which the T cells and MSCs were physically separated by a membrane permeable for soluble factors. The inner hanging cell culture insets with 0.4-μm pore size membrane of a 24-well plate were purchased from Millipore (Billerica, MA, USA). The BM-, AT-, WJ- and PL-MSCs were seeded at 10^5^ cells/well in regular 24-well plates and 24-well Transwell plates containing α-MEM, 10% FBS and 100 U/ml penicillin/streptomycin. After 24 h, 10 μg/ml mitomycin C (MMC; Sigma) were added to inhibit MSC proliferation, and the cells were incubated for 2 h at 37°C followed by 5 extensive washes with medium. A total of 10^5^ T cells/well was added and stimulated with 10 g/ml phytohemagglutinin (PHA; Sigma) and 10 ng/ml interleukin (IL)-2 (Sigma). IL-2/PHA-activated T cells were cultured in the presence or absence of MSCs. The cultures were plated in triplicate and incubated for 5 days before the addition of 5-bromo-20-deoxyuridine (BrdU). After 18 h, proliferation was assessed using the BrdU-Assay kit (Roche Applied Science, Penzberg, Germany) according to the manufacturer’s instructions.

### Statistical analysis

Statistical analyses were performed using SPSS software version 17.0 (SPSS Inc., Chicago, IL, USA). The differences among the treatment groups were analyzed by one-way analysis of variance (ANOVA) and two-way ANOVA with post-hoc analysis using the Dunnett’s test. Parametric data are expressed as the means ± standard deviation (SD). A value of P<0.05 was considered to indicate a statistically significant difference.

## Results

### General characterization of MSCs

**Figure 1:**
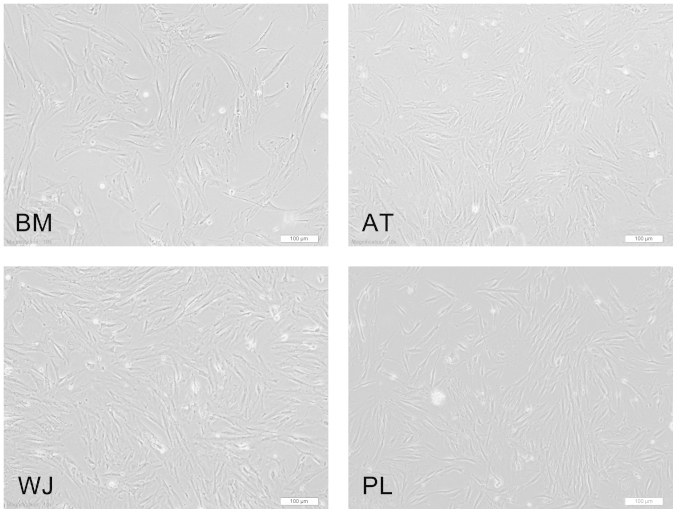
The morphology of mesenchymal stem cells (MSCs) derived from 4 different types of human tissue. MSCs derived from 4 different types of human tissue were isolated and cultured in a 100-mm Petri dish. The cells displayed a fibroblast-like phenotype. Cells in the images are passage 4. Phase contrast magnification, x100. The scale bar represents 100 μm. BM, bone marrow; AT, adipose tissue; WJ, umbilical cord Wharton’s jelly; PL, placenta.

The MSCs derived from 4 different sources (BM, AT, WJ and PL) presented the typical morphology of fibroblastic cells and displayed a high capacity to adhere to the plastic disc (Fig. 1). Following subculture, they showed a strong proliferative ability and they adhered rapidly and expanded without visible changes in either their growth patterns or morphology. The cells reached confluence after approximately 3 days.

### Flow cytometric analysis of MSC makers

**Figure 2:**
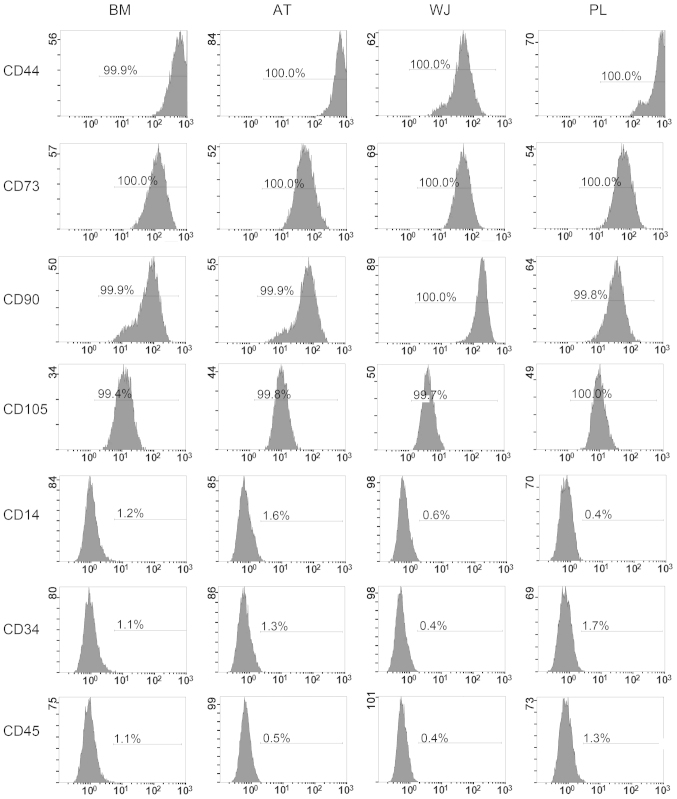
Flow cytometric analysis of mesenchymal stem cell (MSC) makers. Cells at passage 3 were used. BM, bone marrow; AT, adipose tissue; WJ, umbilical cord Wharton’s jelly; PL, placenta. The conjugated fluorescent dyes are: CD44-PE, CD73-PE, CD14-PE, CD34-PE, CD90-FITC, CD45-FITC and CD105-PerCP. The positively stained cells are expressed as a percentage in the middle of the frame. BM, bone marrow; AT, adipose tissue; WJ, umbilical cord Wharton’s jelly; PL, placenta.

The cell-surface antigen profiles of these cells after 3 passages in culture were analyzed by flow cytometry. These cells were strongly positive for MSC-specific surface markers, such as CD44, CD73, CD90 and CD105, but negative for CD14, CD34 and CD45 (Fig. 2). As regards the percentage of cells expressing each angigen, our data showed that >99% of the MSCs from all 4 tissue sources were positive for CD44, CD73, CD90 and CD105, while only ≤1.8% of the cells were positive for CD14, CD34 and CD45 (Fig. 2).

### Adipogenesis and osteogenesis

**Figure 3:**
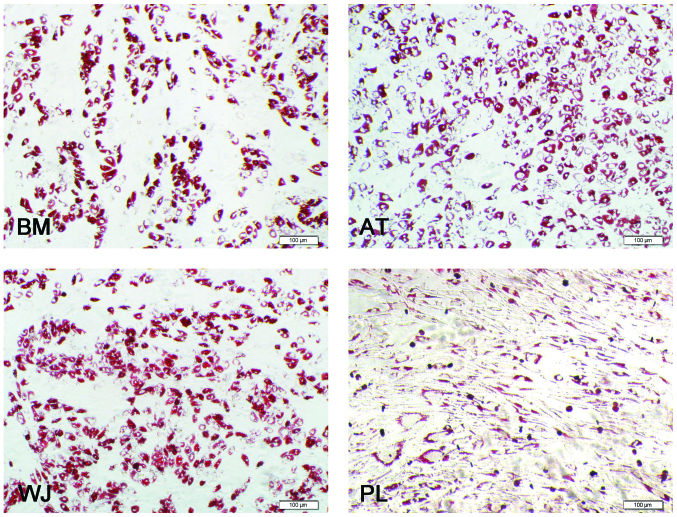
Adipogenesis of mesenchymal stem cells (MSCs). Red color indicates the staining of lipid vesicle-forming adipocytes. All the images have a x100 magnification. The scale bar represents 100 μm. BM, bone marrow; AT, adipose tissue; WJ, umbilical cord Wharton’s jelly; PL, placenta.

**Figure 4:**
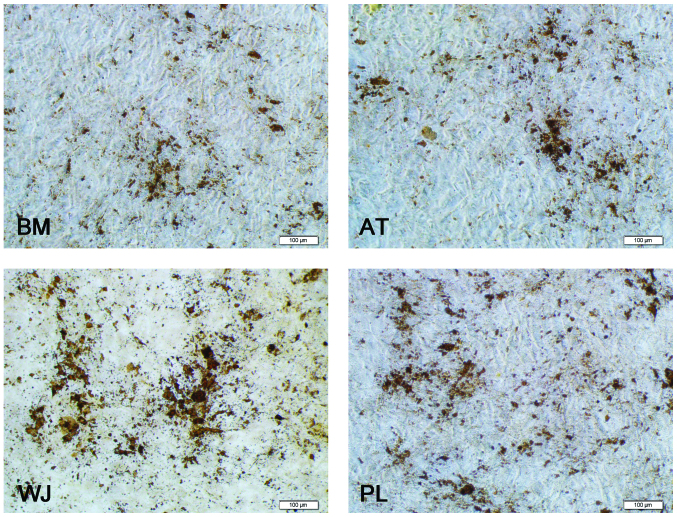
Osteogenesis of mesenchymal stem cells (MSCs). Deposition of calcified nodules was visualized by Alizarin red staining. The ‘red clouds’ demonstrates the mineral deposits in some of the MSCs. All images have a x100 magnification. The scale bar represents 100 μm. BM, bone marrow; AT, adipose tissue; WJ, umbilical cord Wharton’s jelly; PL, placenta.

**Figure 5:**
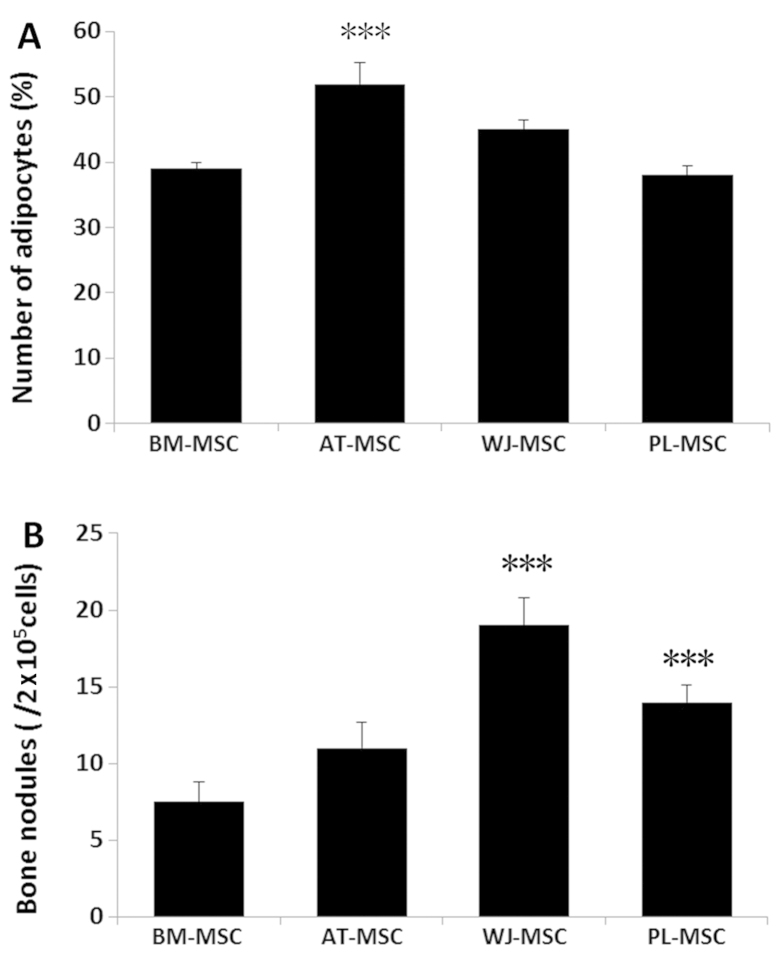
Counting of differentiated adipocytes and bone nodules. Fifteen fields of differentiated adopocytes under a microscope and 9 wells of osteocytes were selected randomly from 3 samples in triplicate. The average of 3 is shown. (A) Adipocyte counting. Stained adipocytes were counted at a magnification of x400. There are 39±1% adipocytes per field from the BM-MSC induction, 52±3.2% from the AT-MSC, 45±1.5% from the WJ-MSC and 38±1.4% from the PL-MSC induction medium. (B) Bone nodule counting. Stained bone nodules were counted at a magnification of x100 in each well of a 12-well plate. A total of 7.5±1.3 bone nodules was counted from the BM-MSC, 11±1.7 from the AT-MSC, 19±1.8 from the WJ-MSC and 14±1.1 from the PL-MSC induction medium. ^***^P<0.001 when compared with BM-MSCs. BM, bone marrow; AT, adipose tissue; WJ, umbilical cord Wharton’s jelly; PL, placenta.

Oil Red O staining revealed that the MSCs were positive for lipid vesicle-forming adipocytes (Fig. 3), and calcium deposits were observed by Alizarin red staining (Fig. 4). For adipogenesis, lipid droplets began to be observed 3 days after the addition of the induction reagents. The time periods by which the lipids appeared were as follows: AT-MSCs, 3 days; BM-MSCs, 6 days; WJ-MSCs and PL-MSCs, 8 days. Subsequently, the size and number of the droplets began to increase until 3 weeks. Although the speed of adipocyte differentiation for the WJ-MSCs and PL-MSCs was slower than that of the BM-MSCs, the final induction ratio differed: the WJ-MSC induction ratio was higher than that of the BM-MSCs, and that of the PL-MSCs was lower than that of the BM-MSCs. For a more detailed comparison, 15 fields at x400 magnification under a microscope were selected and the stained adipocytes were counted. The ratio of differentiated adipocytes from the total cells in the 15 microscopic fields was 52±3.2% for the AT-MSCs, 45±1.5% for the WJ-MSCs, 39±1% for the BM-MSCs and 38±1.4% for the PL-MSCs. Three samples of each population of MSCs were counted (Fig. 5A).

As regards osteogenesis, the cell volume began to increase after 1 week of induction. The shape of the cells gradually became triangular or polygonal and granular matter appeared in the cytoplasm. After 3 weeks of induction, yellow substances accumulated in the MSCs from all 4 tissue sources, particulary in the WJ-MSCs, and gradually, these sediments increased in size to form round or polygonal nodules (Fig. 4). A total of 9 wells from 3 samples of each population of MSCs was analyzed. The average numbers of bone nodules from 1 well were 19±1.8 for the WJ-MSCs (0.01±0.001%), 14±1.1 for the PL-MSCs (0.007±0.0006%), 11±1.7 for the AT-MSCs (0.006±0.0009%) and 7.5±1.3 for the BM-MSCs 0.004±0.0007%). Three samples of each population of MSCs were counted (Fig. 5B). These results demonstrate the multipotent nature of the MSCs. The undifferentiated MSCs cultured in the growth medium did not show any staining for Oil Red O or Alizarin red.

### Proliferative differences in MSCs

**Figure 6:**
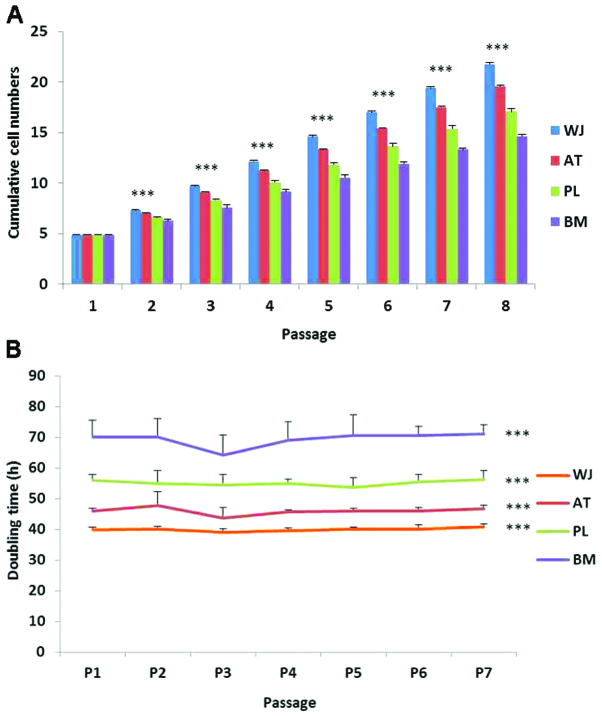
Proliferative potential of mesenchymal stem cells (MSCs). The number of MSCs was counted each time following subculture from 1 to 8 passages. (A) The data are shown as cumulative cell numbers at each passage. Each column represents the mean from 3 MSC populations with standard deviation. (B) The population doubling time was also calculated based on cell counts. ^***^P<0.001 when compared to BM-MSCs. BM, bone marrow; AT, adipose tissue; WJ, umbilical cord Wharton’s jelly; PL, placenta.

During cell proliferation, the MSCs were cultured up to passage 8. The WT-MSCs displayed the highest cumulative cell population followed by the AT-, PL- and BM-MSCs (Fig. 6A). Based on the cell doubling time calculation, the cell doubling time of the WJ-MSCs was approximately 40 h, and that of the BM-MSCs was approximately 70 h (Fig. 6B). The doubling times among the 7 passages did not differ significantly. Thus, the order of the growth rate of the cells was as follows (from the most rapid to the least rapid): WJ-, AT-, PL- and BM-MSCs.

### Expression of immune-related genes in MSCs analyzed by RT-PCR and RT-qPCR

**Figure 7:**
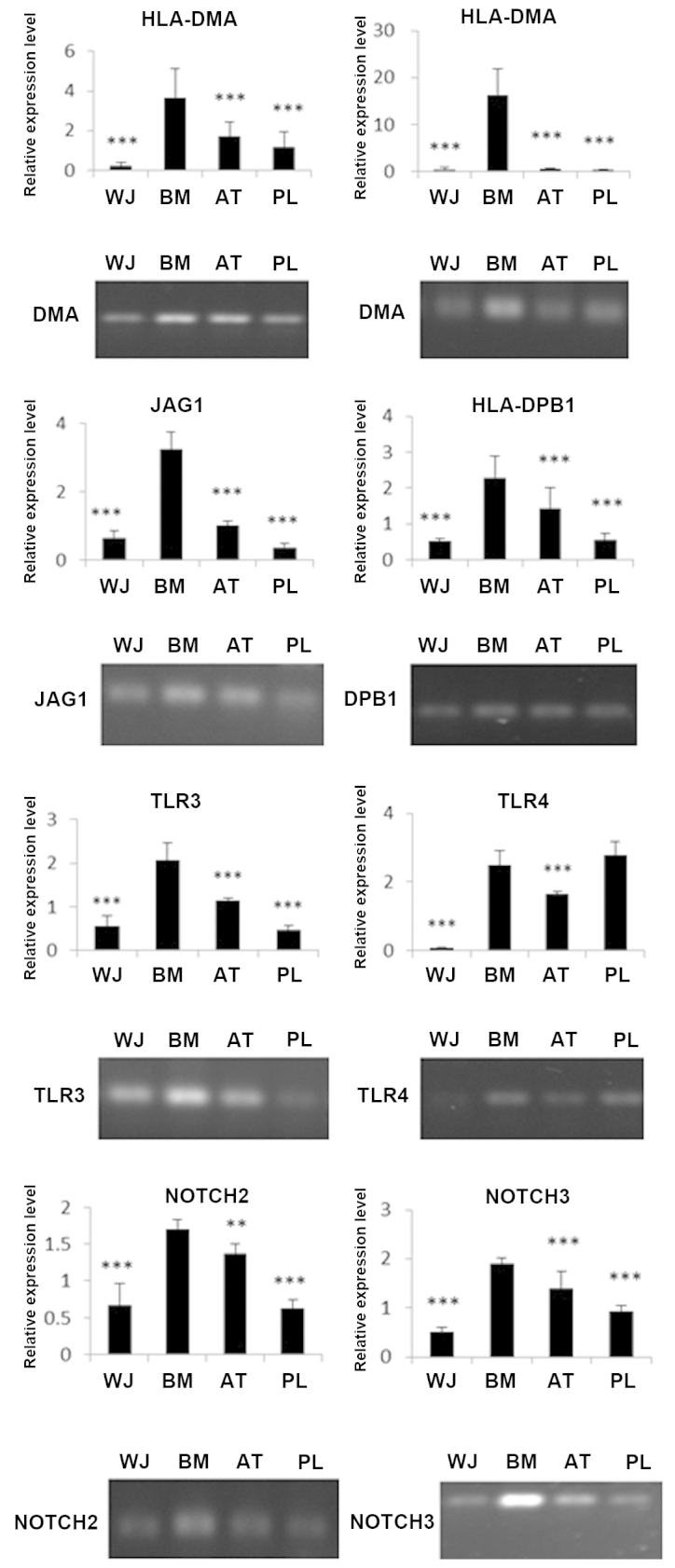
Expression of immune-related genes analyzed by RT-PCR and RT-qPCR. The mRNA level of immune-related genes from the WJ-, AT-, PL- and BM-MSCs was measured by RT-PCR and RT-qPCR. The column charts represent the RT-qPCR data and gel images below the charts represent the RT-PCR data. The data are presented as the means ± SD from 5 independent experiments. ^**^P<0.01 and ^***^P<0.001 when compared to BM-MSCs. BM, bone marrow; AT, adipose tissue; WJ, umbilical cord Wharton’s jelly; PL, placenta.

The MSCs were screened for their surface expression of HLA antigens, co-stimulatory factors and immune tolerance molecules. Through RT-qPCR of HLA-DMA, HLA-DPB1 and HLA-DRA, we found that the BM-MSCs had the highest expression of MHCII molecules than the other MSC populations. The WJ-MSCs had the lowest expression of these molecules among the 4 cell populations. The expression of DMA, DRA and DPB1 in the BM-MSCs was 16-, 36- and 4-fold higher, respectively compared with the WJ-MSCs (Fig. 7). The immune-related genes, JAG1, TLR4, TLR3, NOTCH2 and NOTCH3, were also expressed at different levels. The expression levels of TLR4, TLR3, JAG1, NOTCH2 and NOTCH3 in the BM-MSCs were 38-, 4-, 5-, 3- and 4-fold higher, respectively compared with the WJ-MSCs. The RT-PCR data coincided well with our RT-qPCR results (Fig. 7).

### MSC inhibition of T cell proliferation

**Figure 8:**
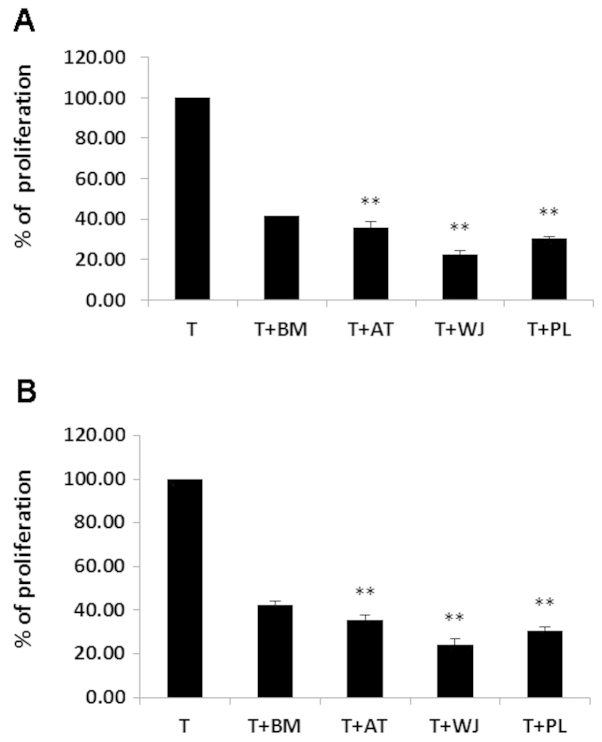
T cell proliferation was inhibited by mesenchymal stem cells (MSCs) in co-culture. The first column is the T cell only control. (A) Cell to cell contact co-culture. The T cell proliferation ratio was reduced to 41.4±3.2%, 35.5±1.7%, 22.6±0.9% and 30.4±1.2%, respectively by MSCs derived from bone marrow (BM), adipose tissue (AT), umbilical cord Wharton’s jelly (WJ) and placenta (PL). (B) Transwell co-culture. The T cell proliferation ratio was reduced to 42.6±1.6%, 35.7±1.8%, 24±2.7% and 30.8±1.7%, respectively by co-culture with BM-MSCs, AT-MSCs, WJ-MSCs, and PL-MSCs. The data are expressed as the means ± SD from 5 independent experiments. The inhibitory effect of the AT-, WJ- and PL-MSC co-culture groups on T cell proliferation was more prominent than that of the BM-MSC group. ^**^P<0.01 when compared to the control.

PHA/IL-2-activated T cells were incubated on a layer of MSCs derived from BM, AT, WJ and PL tissue. T cell proliferation decreased significantly following co-culture with the MSCs. The ratio of growth retardation of the T lymphocytes in the contact culture was: BM-MSCs, 41.4±3.2%; AT-MSCs, 35.5±1.7%; WJ-MSCs, 22.6±0.9% and PL-MSCs, 30.4±1.2% when compared to the T cells cultured alone. The results from Transwell culture were similar to those of the contact culture (P>0.05). The inhibitory effects of the AT-MSCs, WJ-MSCs and PL-MSCs on T cell proliferation were more prominent than those of the BM-MSCs (P<0.01). The inhibitory effects of the WJ-MSCs on T cell proliferation were the most prominent (P<0.01; Fig. 8).

## Discussion

BM is the earliest tissue source for MSC extraction and represents the most commonly used source for MSCs in clinical treatments. However, there are some limitations in using BM-MSCs due to the high degree of viral exposure and the significant decrease in cell number and proliferation/differentiation capacity with the increasing age of the donor (29,30). It is also a painful and invasive procedure to obtain BM from patients for MSC use. These disadvantages have encouraged researchers to find a better tissue source to replace BM for isolating MSCs. AT-MSCs have gained increasing attention given the increasing problem posed by obesity in recent years. In addition, AT-MSCs have a similar proliferative ability and differentiation potential to BM-MSCs (31). MSCs derived from human WJ of the umbilical cord also have the ability to differentiate into multiple lineages, given the appropriate conditions. Kobayashi *et al* also revealed that matrix cells from the WJ showed similar characteristics with those of MSCs derived from BM (32). In addtion, progenitor cells or stem cells may be isolated from the human term placenta (33). Human BM, AT, WJ and PL are the main sources of MSCs, and these types of MSCs are frequently used in both experimental and clinical studies. Although MSCs derived from these 4 sources share global properties, such as morphology, plastic adherence and multilineage potential, they diverge in terms of their phenotypes. Up to now, there have been no detailed studies comparing these MSCs derived from different sources. However, we do know that they have different proliferative, differentiation and immune properties.

In this study, we performed a side-by-side comparison of 4 populations of MSCs derived from BM, AT, WJ and PL simultaneously. We observed significant differences in the proliferative potential among the 4 populations of MSCs; the WJ-MSCs exhibited the highest growth rate. The growth curve showed that the proliferative capacity of the WJ-, AT- and PL-MSCs was significantly greater than that of the BM-MSCs. These data coincide with those of previous studies (34–37).

Some believe that the growth differences may not reflect compartment-specific characteristics, but most probably reflect differences in newborn and adult donors (38). Alternatively, differences in growth rate may reflect culture heterogeneity with variable proportions of self-renewal versus lineage-committed cells in different stromal cell compartments (39,40). In this study, the order of the proliferation rate from the fastest to the slowest was: WJ-MSCs, AT-MSCs, PL-MSCs and BM-MSCs. Basically, these data follow the age trend except, that the AT was obtained from older individuals than those from which the PL was obtained (6).

MSCs derived from different tissues have been demonstrated in a large number of studies to differentiate into cells in the mesodermal lineages, such as osteoblasts and adipocytes (41–44). Our results demonstrated that there are quantitative differences between different populations of MSCs with respect to their differentiation potentials. Our data demonstrated that of the 4 different populations of MSCs, the AT-MSCs possessed the strongest adipogenic potential followed by the WJ-MSCs, BM-MSCs and PL-MSCs. Although the BM-MSCs differentiated into adipocytes earlier than the WJ-MSCs, the final adipocyte ratio was lower in the BM-MSCs than in the WJ-MSCs. Therefore, our data indicate that the WJ-MSCs have a greater adipogenic potential than the BM-MSCs.

As regards osteogenesis, the results revealed that the WJ-MSCs had the greatest potential in osteogenesis followed by the PL-MSCs, AT-MSCs and MB-MSCs. Since the WJ and PL tisssues were obtained from individuals who were much younger than the individuals the AT and BM tissues were obtained from, we predicted that the MSCs derived from younger tissue sources would differentiate more easily into osteoblasts that those obtained from older tissue sources. Baksh *et al* (35) also demonstrated that the osteogenetic potential of WJ-MSCs was higher than that of BM-MSCs.

It is a widely accepted fact that MSCs have immunosuppressive and immunomodulatory functions. Due to their ability to regulate immune responses, MSCs are a potential candidate for treating a wide range of immune-mediated diseases (45,46). Therefore, comparing the immune properties of MSCs derived from different sources may have great value in selecting the one which would be most effective in clinical treatment.

HLA-DRA is one of the HLA class II α chain paralogues. This class II molecule is a heterodimer consisting of an α and a β chain, both anchored in the membrane. It plays a central role in the immune system by presenting peptides derived from extracellular proteins, such as HLA-DPB1. HLA-DMA is an antigen processing and presentation-related gene. Our data demonstrated that the WJ-MSCs expressed the lowest levels of the HLA class II genes, HLA-DRA, HLA-DPB1 and HLA-DMA, than any other 3 populatinos of MSCs, while the BM-MSCs expressed the highest level of these 3 genes. These data suggest that the WJ-MSCs may be the most effective population of MSCs for clinical application.

We also analyzed the mRNA expression of JAG1, TLR4, TLR3, NOTCH2 and NOTCH3 since they are expressed in MSCs and are closely related to immune response (47). Our data demonstrated that the expression levels of TLR4, TLR3, JAG1, NOTCH2 and NOTCH3 in the BM-MSCs were 38-, 4-, 5-, 3- and 4-fold higher, respectively compared with the WJ-MSCs. These data suggest that WJ-MSCs may have the strongest immunosuppressive potential and produce only a slight, if any immune response.

It has been shown that MSCs possess the intrinsic homing ability to migrate to injured tissues and actively participate in tissue repair. In addition, MSCs possess the unique ability to suppress immune responses, both *in vitro* (48) and *in vivo* (49). In order to further assess the immune characteristics of MSCs, we examined their direct effect on mitogen (PHA/IL-2)-activated T cell proliferation using co-culture experiments. PHA/IL-2-activated T cells were incubated on a layer of MSCs derived from BM, AT, PL and WJ. The results demonstrated that the suppressive effects of the MSCs derived from AT, PL and WJ on T cell proliferation were more prominent than those of the MSCs derived from BM. Our results are in accordance with those of a previous study (50). Our data demonstrated that the inhibitory effect of WJ-MSCs on T cell proliferation was the most prominent. Previous studies have indicated that MSCs exert immunosuppressive effects either through direct cell-cell contact or by soluble factors (51). Studies have implicated contact-dependent mechanisms, including the expression of B7H1 on MSCs (51). Other studies have reported that soluble factors secreted by MSCs, or by immune cells in response to MSCs, play a major role in MSC-mediated immune suppression (52). To determine this, we performed co-culture experiments using both the contacted mix culture and the Transwell system in which T cells and MSCs were physically separated by a membrane permeable for soluble factors. Our data revealed that the inhibitory effects of MSCs on T lymphocytes were similar from both the contact culture and Transwell culture. This illustrates that soluble factors may be involved in the immunosuppressive properties of MSCs.

In this study, we demonstrate that the proliferative potential of WJ-MSCs is higher than that of AT-MSCs, PL-MSCs and BM-MSCs. WJ-MSCs have the strongest osteogenic ability followed by the PL-MSCs, AT-MSCs and BM-MSCs. WJ-MSCs have the weakest expression of MHC II genes, while BM-MSC have the highest. WJ-MSCs have the weakest expression of the immune-related genes, TLR4, TLR3, JAG1, NOTCH2 and NOTCH3. Furthermore, WJ-MSCs have the most prominent suppressive effect on T lymphocytes among the 4 populations of MSCs in the co-culture experiments. Therefore, WJ-MSCs may be one of the best sources of MSCs for tissue regeneration in future clinical application.

## Figures and Tables

**Table I:** Primers used for RT-qPCR.

Gene name	Primers	Sequences	Product size (bp)
HLA-DMA	Forward	AAAATCCCGGTGTCCAGAG	167
	Reverse	GTAGGCCCAAATCCTTCCA	
HLA-DRA	Forward	CCTGACTGTGGGTCTGGTG	81
	Reverse	CGTTCTGCTGCATTGCTTT	
HLA-DPB1	Forward	CCTGGTGATGCTGGAAATG	105
	Reverse	GACTGTGCCTTCCACTCCA	
JAG1	Forward	CGGCCTCTGAAGAACAGAAC	82
	Reverse	TCACCAAGCAACAGATCCAA	
TLR4	Forward	TTTCACCTGATGCTTCTTGCT	103
	Reverse	TCCTTACCCAGTCCTCATCCT	
TLR3	Forward	AGGATTGGGTCTGGGAACA	95
	Reverse	AAAAACACCCGCCTCAAAG	
NOTCH2	Forward	TGGGCTACACTGGGAAAAAC	107
	Reverse	TAGGCACTGGGACTCTGCTT	
NOTCH3	Forward	CTCATCCGAAACCGCTCTAC	101
	Reverse	TCTTCCACCATGCCCTCTAC	
